# Effectiveness of Rotarix ^®^ vaccine in Africa in the first decade of progressive introduction, 2009-2019:  systematic review and meta-analysis

**DOI:** 10.12688/wellcomeopenres.16174.2

**Published:** 2020-09-24

**Authors:** Nickson Murunga, Grieven P. Otieno, Marta Maia, Charles N. Agoti

**Affiliations:** 1 Epidemiology and Demography Department, Kenya Medical Research Institute (KEMRI)-Wellcome Trust Research Programme, Kilifi, Kenya; 2 Department of Public Health, School of Health and Human Sciences, Pwani University, Kilifi, Kenya; 3 Centre for Tropical Medicine and Global Health, Nuffield Department of Medicine, University of Oxford, Old Road Campus Roosevelt Drive, Oxford, OX3 7FZ, UK

**Keywords:** Rotavirus, vaccine effectiveness, systematic review, meta-analysis, Africa

## Abstract

**Background:** Randomized controlled trials of licensed oral rotavirus group A (RVA) vaccines, indicated lower efficacy in developing countries compared to developed countries. We investigated the pooled effectiveness of Rotarix ^® ^in Africa in 2019, a decade since progressive introduction began in 2009.

**Methods:** A systematic search was conducted in PubMed to identify studies that investigated the effectiveness of routine RVA vaccination in an African country between 2009 and 2019. A meta-analysis was undertaken to estimate pooled effectiveness of the full-dose versus partial-dose of Rotarix ^®^ (RV1) vaccine and in different age groups. Pooled odds ratios were estimated using random effects model and the risk of bias assessed using Newcastle-Ottawa scale. The quality of the evidence was assessed using GRADE.

**Results:** By December 2019, 39 (72%) countries in Africa had introduced RVA vaccination, of which 34 were using RV1. Thirteen eligible studies from eight countries were included in meta-analysis for vaccine effectiveness (VE) of RVA by vaccine dosage (full or partial) and age categories. Pooled RV1 VE against RVA associated hospitalizations was 44% (95% confidence interval (CI) 28-57%) for partial dose versus 58% (95% CI 50-65%) for full dose. VE was 61% (95% CI 50-69%), 55% (95% CI 32-71%), 56% (95% CI 43-67%), and 61% (95% CI 42-73%) for children aged <12 months, 12-23 months, <24 months and 12-59 months, respectively.

**Conclusion:** RV1 vaccine use has resulted in a significant reduction in severe diarrhoea in African children and its VE is close to the efficacy findings observed in clinical trials. RV1 VE point estimate was higher for children who received full dose than those who received partial dose, and its protection lasted beyond the first year of life.

## Introduction

Globally, rotavirus group A (RVA) is a leading cause of severe dehydrating acute diarrhoea in children aged <5 years ^1^. In 2016, approximately 117 million episodes of rotavirus-associated diarrhoea occurred in sub-Saharan Africa, ~104,000 of which were fatal ^2^. Rotavirus vaccination programmes are considered the most effective control measure for RVA disease ^3, 4^ and six oral vaccines (RotaTeq® (RV5), Rotarix® (RV1), Rotavac®, Rotavin-mi®, Lanzhou Lamb and Rotasiil®) have been licensed ^5^. In 2009, the World Health Organization recommended inclusion of two licensed RVA vaccines (Rotarix®, GlaxoSmithKline Biologicals, Belgium; and RotaTeq®, Merck, USA) into routine national immunization programmes (NIP) of all countries ^6^. By July 2020, 74% of African countries (40 out of 54) had introduced an RVA vaccine into their NIP compared to the global tally of 107 out of 194 countries (55%) ^7^. Of the 40 African countries with the RVA vaccine, 35 (88%) were using the RV1 vaccine.

Randomised controlled trials (RCTs) investigating the efficacy of oral RVA vaccines in sub-Saharan Africa showed a modest performance (50–80% efficacy against severe disease) compared to results from industrialized countries (90–100% efficacy) ^8, 9^. Despite this discrepancy, use of RVA vaccines in developing countries was encouraged on the basis of the expected absolute impact on the high RVA disease burden in low-income setting ^10^. Post-vaccine introduction, a number of African countries have reported on RVA vaccine impact and effectiveness against rotavirus gastroenteritis (RVGE) and all-cause diarrhoea related hospitalisations ^11^. Vaccine effectiveness is similar to vaccine efficacy but is measured in the context of routine real-world use of the vaccine to quantify the reduction in disease among those who are vaccinated compared to unvaccinated persons. Vaccine impact measures the absolute reduction in disease at population level following the introduction of the vaccine and is determined by from vaccine effectiveness, vaccine coverage, and any herd effect ^12^. There have been expert reviews discussing the impact of RVA vaccine in African countries ^13– 15^ and systematic review and meta-analysis conducted focusing on the prevalence of rotavirus infections pre- and post-vaccine introduction ^16^. In this paper, we present a systematic review and meta-analysis of vaccine effectiveness of rotavirus vaccination programmes in Africa focusing on partial dose versus full dose and effectiveness stratified by age categories.

## Methods

### Systematic search

We conducted a systematic search in PUBMED database for articles on research conducted in African populations from January 2009 to December 2019 focusing on the rotavirus vaccination programme and adhered to PRISMA guidelines ( *Extended data*: Supplementary File One, Supplementary Table 1) ^17^. Publications were identified using combinations of the following key search terms: “Rotavirus”, “effectiveness”, “success”, “impact”, “effect”, “potency”, “performance”, “vaccine”, “Rotarix”, “Rotateq” and names of all 54 African countries. We restricted our search to articles published in English (see *Extended data*: Supplementary File One, Supplementary Text 1 for details) ^17^. Two reviewers screened the outputs identified from the searches for appropriate articles and from references of the relevant published articles to identify additional articles for possible inclusion into the analysis. The final included articles were based on agreement between the two reviewers. A third reviewer resolved any discrepancies. Information on RVA vaccine introduction status for each country and impact evaluation were inferred from VIEW-hub ^18^.

### Inclusion criteria and outcomes

This analysis focuses on the Rotarix® (RV1) Vaccine, which is given to infants as two doses at 6 and 10 weeks of life. We aimed to include articles published from any African country that administers RV1 vaccine as part of the NIP. Observational studies (case-control) reporting on the effectiveness of RVA vaccine among children aged <5 years in their country, on RVGE or other acute gastroenteritis (AGE) hospitalization between 2009 and 2019 were included. Outcomes of interest included effectiveness RVA vaccine against hospitalization from RVGE for full dose, partial dose and, effectiveness stratified by age categories. Randomized controlled trials, review articles, editorials and conference papers were excluded from this analysis ( *Extended data*: Supplementary File One, Supplementary Text 2) ^17^.

### Data extraction

The following details were extracted from the eligible studies; study design, sample size, country, vaccine type, age groups, cases vaccinated, controls vaccinated, reported vaccine effectiveness and 95% confidence intervals (CIs). Extracted data was entered into data collection forms created in Microsoft Excel (Extended data: Supplementary File Two) ^17^.

### Assessment of risk of bias

The Newcastle–Ottawa scale (NOS) was used to assess the risk of bias (ROB) among the case-control studies ^19^. The NOS was used to evaluate the selection of participants, comparability of study groups, and the ascertainment of exposure or outcome of interest.

A study was assigned a maximum of 9 points based on selection (4 stars), comparability (2 stars) and exposure (3 stars), for a maximum of 9 points, by using a star allocation scheme according to the coding manual developed by collaboration between Universities of Newcastle and Ottawa ^20^. Studies scoring zero in any of the categories were classified as having high ROB. Studies scoring 1 point in any of the categories were classified as having moderate ROB, and those scoring 2 points or more in all categories were classified as having low ROB.

### Data management and analysis

Meta-analysis was stratified (full and partial dose) for RV1 vaccine effectiveness and by age categories (<12 months, 12–23 months, <24 months, and 12–59 months). We used study reported vaccine effectiveness (VE) estimates $\sqrt{VE=(1-OR)*100\%}$ to obtain the respective **$\sqrt{OR=(1-VE/100)}$** and the respective log transformed odds ratio (OR). A random effects model was used to estimate the pooled VE while accounting for variations of the true effect size due to varying geographical settings of the studies.

Heterogeneity was assessed by the chi-squared test for heterogeneity and quantified by *I ^2^* index [(Q-df) / Q x 100 %] where Q is the Cochran’s heterogeneity statistics and the degrees of freedom (df). *I ^2^* values of 25–49%, 50–74% and >=75% were categorized as low, moderate, and high heterogeneity respectively ^21^. Forest plot was used to present the pooled ORs with their corresponding 95% CI. To check for publication bias, funnel plot was used, and Eggers test employed to assess funnel plot asymmetry. All statistical analyses were conducted using STATA version 15.1 (StataCorp College Station, Texas), and where applicable admetan package was used ^22^.

### Quality of the evidence

Two reviewers independently assessed the quality of evidence using the Grading of Recommendation, Assessment, Development, and Evaluation (GRADE) approach ^23^. Quality of evidence was graded as high, moderate, low, or very low. GRADE starts with a baseline rating of ‘high quality of evidence” for RCTs, and “low quality of evidence” for non-RCTs ^24^. Given that only observational studies were included in this review we assessed the quality of evidence starting-off as “low quality” and downgraded or upgraded accordingly. Reasons for downgrading included high risk of bias, inconsistency or heterogeneity, indirectness of the findings, imprecision of the point estimates, and publication bias. The quality of evidence was upgraded if data showed a large effect, a dose-response effect, or if all the plausible residual confounding would reduce the demonstrated effect or would suggest a spurious effect if no effect was observed.

## Results

### Search outcome

**Figure 1.**
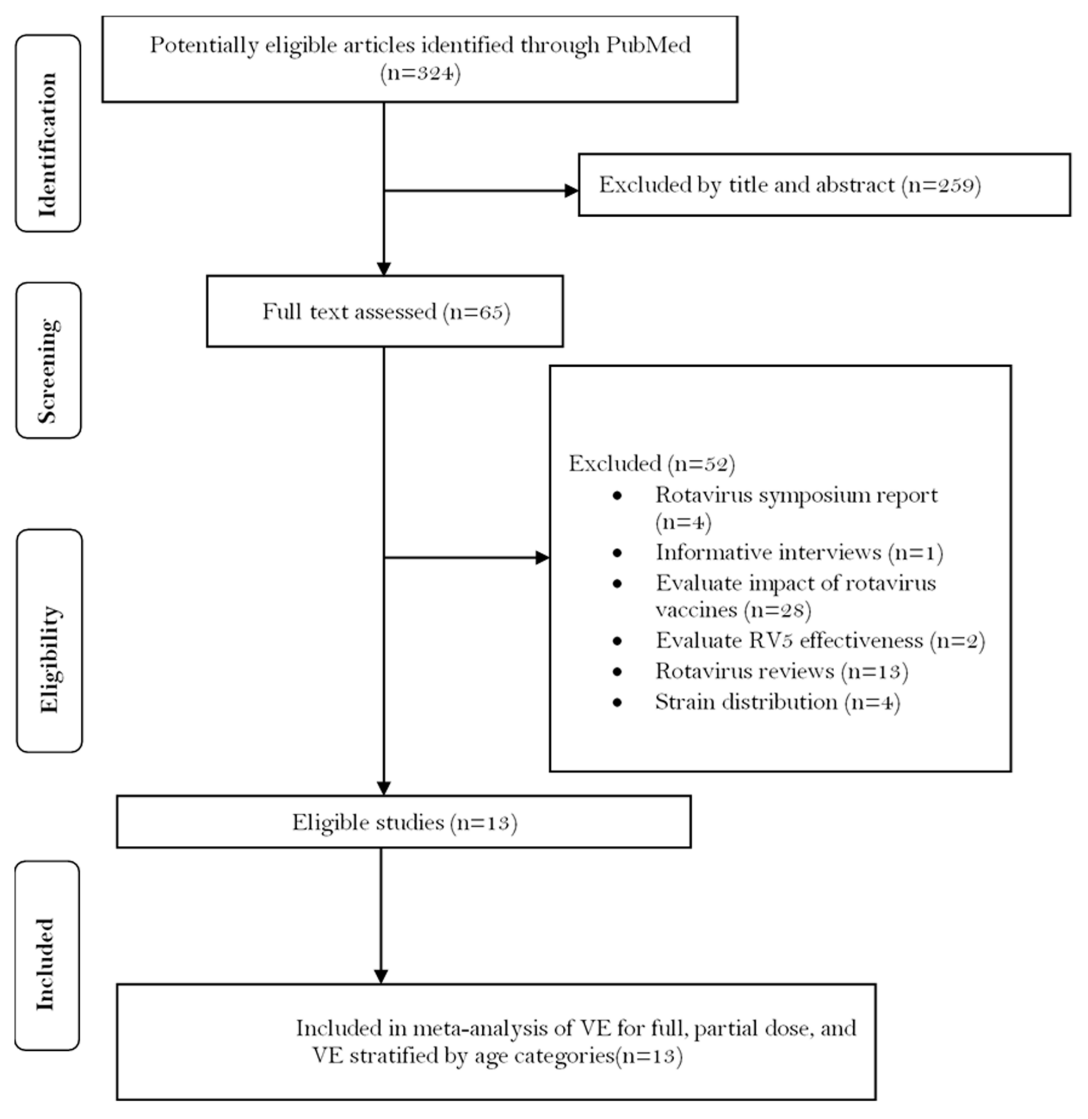
Identification of studies included in the systematic review. VE=Vaccine effectiveness.

A total of 324 published articles were identified based on our defined search criteria. Of these, 13 met our inclusion criteria for the meta-analysis ( Figure 1 and Table 1). All the 13 studies reported the full dose vaccine effectiveness and five studies also reported on effectiveness of partial dose of RV1 vaccine as well. The included studies originated from eight countries. The large majority of studies were excluded from the analysis after screening the title and abstract (n=259). Fifty-two studies were excluded after full-text screening. Of these, 28 were due to evaluation of the impact of RVA vaccines, 13 were systematic reviews, four were rotavirus symposium report, and rotavirus strain distribution respectively, two were evaluating the effectiveness of RVA pentavalent vaccine (RotaTeq®), and one was an informative interview ( Figure 1; see *Extended data*: Supplementary File One, Supplementary Text 2 for details) ^17^. All the studies included in this review used enzyme immunoassay (EIA) to identify rotavirus infection (Extended data: Supplementary File Two) ^17^.

**Table 1.** Characteristics of case-control studies included in analysis of vaccine effectiveness.

Study	Country	Age (m/w)	Sample size	Cases vaccinated (N/Total)		Control vaccinated (N/Total)		Adjusted VE (95% CI)	
				Partial Complete		Partial Complete		Partial dose Full dose	
Beres *et al.* 2016 ^27^	Zambia	>=6m	316	1/18	8/18	28/298	182/298	62 (-261 to 96)	56 (-34 to 86)
Bar-zeev *et al.*, 2015 ^28^	Malawi	<12m	392	NR	81/109	NR	234/283	NR	64 (24-83)
Armah, *et al.* 2016 ^25^	Ghana	6-<24m	655	NR	196/207	NR	426/448	NR	18 (-81 to 63)
Bar-zeev *et al.*, 2016 ^29^	Malawi	<60m <12m 12–23m	933 634 272	NR NR NR	241 167 71	NR NR NR	692 467 201	NR NR NR	58.3 (20.2-78.2) 70.6 (33.6-87.0) 31.7 (−140.6 to 80.6)
Plattis-mills *et al.*, 2017 ^30^	Tanzania	<60m	220	NR	57/71	NR	121/149	NR	74.8 (-8.2 to 94.1)
Abeid, *et al.* 2017 ^31^	Tanzania	5–23m 5–11m 12–23m	691 474 217	NR NR NR	157/179 94/107 63/72	NR NR NR	480/512 345/367 135/145	N/R N/R N/R	57(14-78) 56(-2 to 81) 57(-30-86)
Ganstanaduy *et al.*, 2016 ^32^	Botswana	>=4m 4–11 ≥12	610 425 185	37/242 26/162 11/80	162/242 108/162 54/80	51/368 38/368 13/105	288/368 202/368 86/105	48(1-72) 42(-23 to 73) 63(-33 to 90)	54(23-73) 52(8-75) 67(8-89)
Groome, *et al.* 2014 ^33^	South Africa	18w–23m 18w-11m 12–23m	1974 1336 638	126/540 92/389 34/151	278/540 207/389 71/151	334/1434 231/947 103/487	856/1434 567/947 289/487	40(16-57) 39(9-59) 40(-7-66)	57(40-68) 54(32-68) 61(35-77)
Mujuru *et al.* 2019 ^26^	Zimbabwe	6–11m >=12m	1467 2121	371/398 482/505	NR NR	NR NR	NR NR	NR NR	61 (21-81) -48 (-148 to 11)
Mokomane *et al.* 2018 ^34^	Botswana	>=4m	610	37/242	162/242	51/368	288/368	48 (1-72)	54 (23-73)
Jani B *et al.* 2018 ^35^	Tanzania	5–23m	609	NR	110/119	NR	470/490	NR	49 (-30 to 80)
Khagayi *et al.* 2020 ^36^	Kenya	<60m <12m >=12m	509 273 175	7/40 NR NR	51/83 33/55 18/28	41/110 NR NR	308/365 184/218 124/147	54 (-20 to 83) NR NR	64 (35-80) 67 (30-84) 72 (10-91)
Bennett A *et al.* 2018 ^37^	Malawi	<60m <12m 12–23m	1318 1318 1318	NR NR NR	275/1019 190/696 78/285	NR NR NR	NR NR NR	NR NR NR	61.89 (28.04–79.82) 74.88 (44.59-88.61) 31.69 (-139.03 to 80.48)

VE: Vaccine effectiveness; NR: Not recorded; CI: Confidence interval; Partial dose: One dose of Rotarix; Full dose: Two doses of Rotarix; age (m/w): Age in months or weeks.

### Risk of bias in the included studies

ROB in the included studies was assessed, and none was found to have a high risk of bias. The assessment using NOS was based on selection, comparability, and exposure. With regard to selection; most studies had adequate selection and representativeness of cases, controls were selected within the same population as cases, and it was difficult to ascertain if the history of rotavirus among the control was considered. With regard to comparability; confounding was controlled through adjusting for age, date of birth, and month and year of admission in the analysis for the majority of the studies. Lastly, exposure was ascertained through the vaccination cards held by a guardian or parent. It was difficult to ascertain whether the non-response rate was considered in the analysis of most studies (see *Extended data*: Supplementary File Three for details) ^17^.

### Pooled vaccine effectiveness of Rotarix® (RV1) vaccine

**Figure 2.**
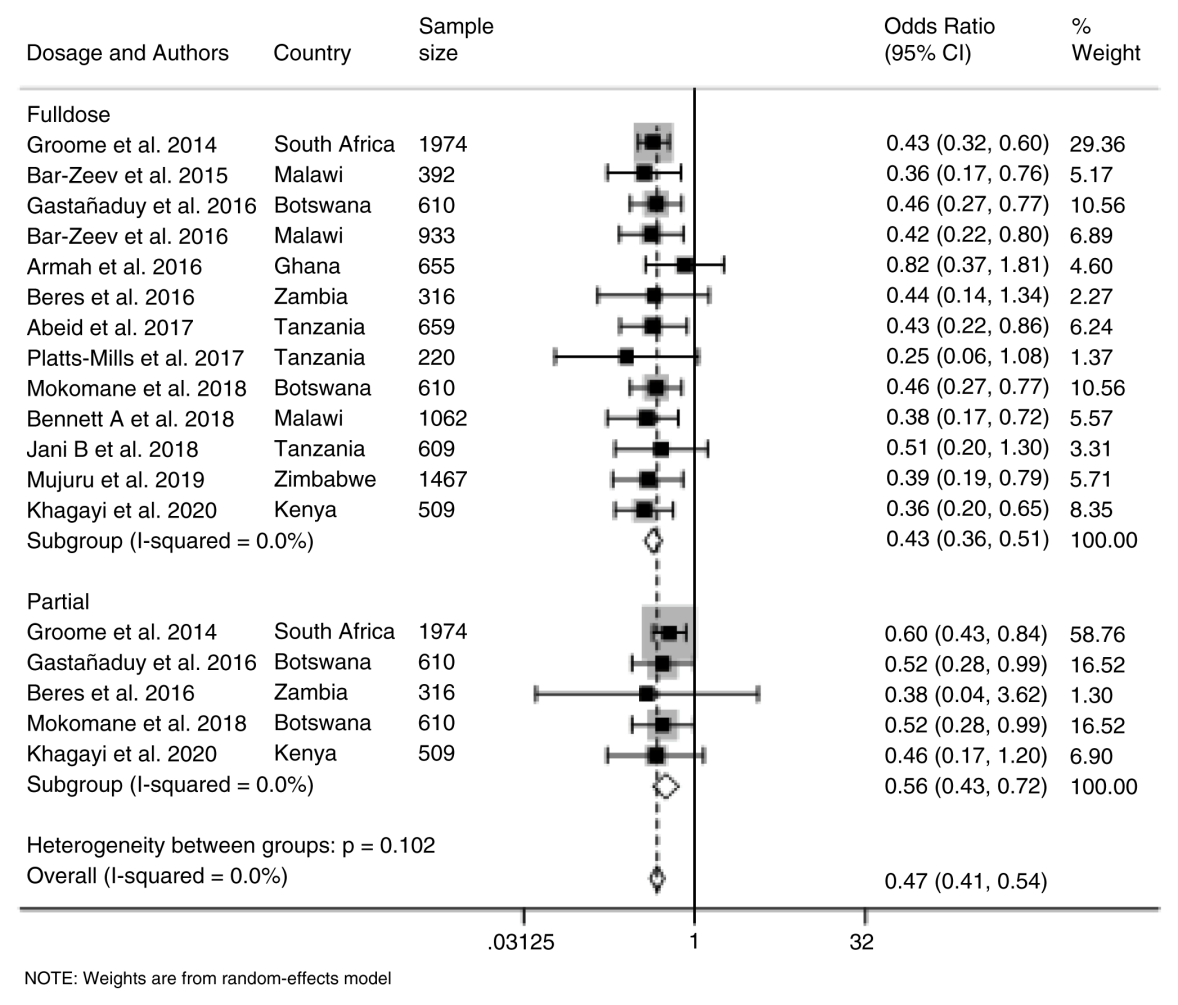
Forest plot of vaccine effectiveness of full and partial doses against hospitalization for rotavirus gastroenteritis. Studies are plotted starting with the earliest published to the recent. Each study is represented by a black box and a horizontal line, which correspond to the odds ratio and 95% CI, respectively. The vertical line in the middle corresponds to an odds ratio of 1.0. The diamond represents the overall pooled odds ratio with the 95% CI given by its width. I-squared shows the degree of heterogeneity with p-value indicating whether there was statistically significant heterogeneity between the studies and among the groups. Fulldose stands for two doses of the Rotarix while Partial represents one dose of Rotarix.

**Figure 3.**
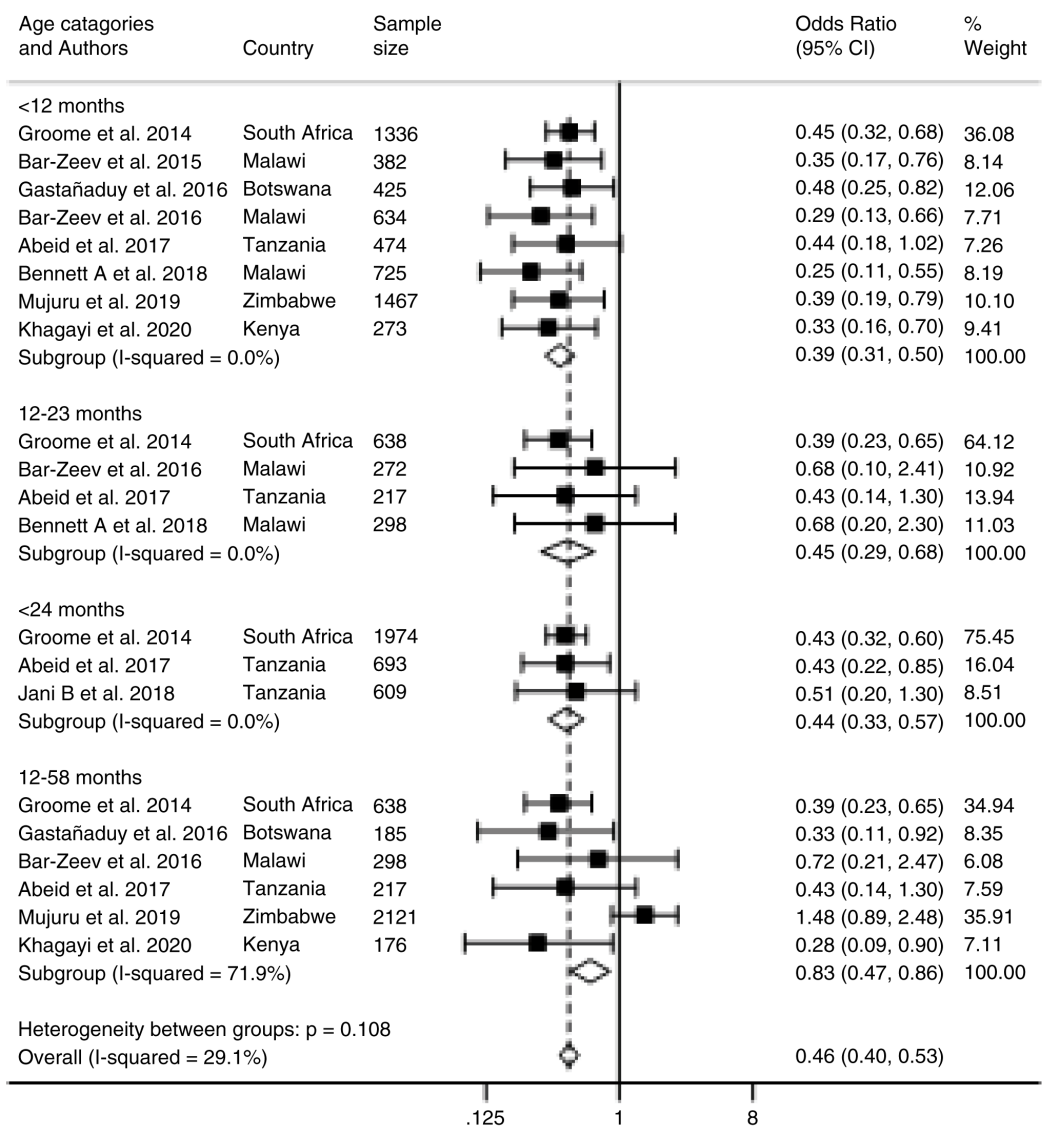
Forest plot of vaccine effectiveness against hospitalization for rotavirus gastroenteritis stratified by age groups. Studies are plotted starting with the earliest published to the recent. Each study is represented by a black box and a horizontal line, which correspond to the odds ratio and 95% CI, respectively. The vertical line in the middle corresponds to an odds ratio of 1.0. The diamond represents the overall pooled odds ratio with the 95% CI given by its width. I-squared shows the degree of heterogeneity with p-value indicating whether there was statistically significant heterogeneity between the studies and among the groups.

**Figure 4.**
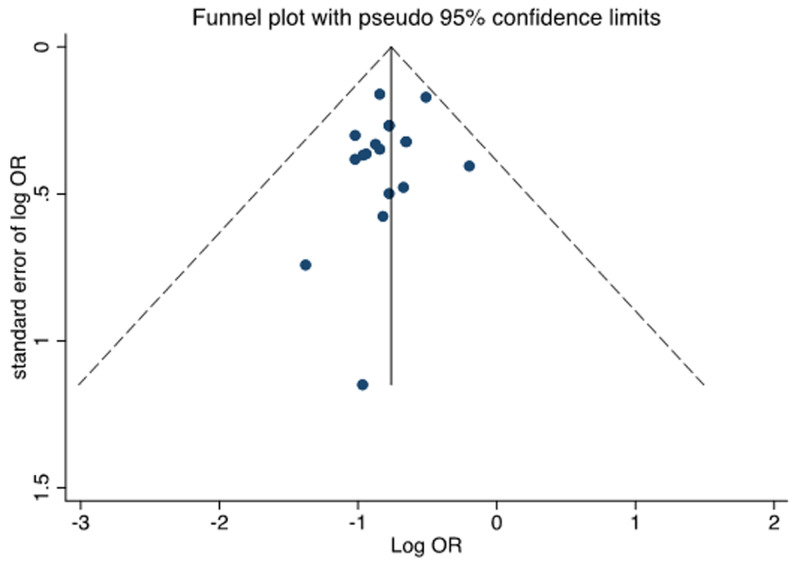
Funnel plot to assess publication bias among studies evaluating effectiveness of RV1 vaccine against hospitalization for laboratory-confirmed rotavirus gastroenteritis. The triangle represents the estimates of the included studies that reported on the effectiveness of full and partial dose of RV1 vaccine. The log of the odds ratio is plotted on the horizontal axis, against the standard error of the log odds ratio. The vertical line in the funnel plot indicates the random effect summary estimate and the sloping two lines indicate the expected 95% CIs for a given SE.

Pooled RV1 VE against rotavirus-associated hospitalization was estimated as 44% (95% CI 28–57%) among children who received partial dose versus 57% (95% CI 49–64%) among children who received the full dose. There was no statistical evidence of heterogeneity for studies reporting estimates for both full ( *I ^2^* = 0.0, *p* = 0.98) and partial dose ( *I ^2^* = 0.0, *p* = 0.97) ( Figure 2). When stratifying by age (<12 months, 12–23 months, <24 months and 12–59 months), full dose of RV1 had an effectiveness of 61% (95% CI 50–69%), 55% (95% CI 32–71%), 56% (95% CI 43–67%), and 37% (95% CI 14–53%), respectively ( Figure 3). Statistically significant heterogeneity ( *I ^2^* = 71.9 %, *p* = 0.003) was observed for 12–59 months age category. No heterogeneity was observed in other age stratification ( *I ^2^* = 0.0 %, p ** >0.05). Four and three studies reported VE for children aged 12–23 and <24 months, respectively. These estimates should however be interpreted with caution due to few numbers of studies used. Publication bias was not observed among the studies that reported on effectiveness of RV1 vaccines ( Figure 4). This was supported by eggers regression intercept (-0.43; 95 % CI -1.2 to 0.37; p=0.273).

### Sensitivity analysis

Sensitivity analysis was carried out by excluding Armah *et al.* 2016 ^25^ in our meta-analysis of full and partial dose VE. This study reported full dose vaccine effectiveness of 18% (95% CI -81–63%). This was due to high vaccine coverage (93–100%) immediately after its introduction in Ghana making it difficult to arrive at robust VE estimate ^25^. VE estimate for full dose by excluding this study in our meta-analysis was 58% (95% CI 50–65%) ( *Extended data*: Supplementary File One, Supplementary Figure 1) ^17^. More so, sensitivity analysis was carried out to ascertain the source of high heterogeneity when meta-analysis was stratified by age categories. A study by Mujuru *et al.* 2019 ^26^ was dropped from analysis because the author stated that VE estimate for this aged group lacked precision and was non-significant ^26^. VE estimate for full dose after this study was dropped from analysis was 61% (95 % CI 42–73%) and no heterogeneity was observed in any of the age stratification ( *Extended data*: Supplementary File One, Supplementary Figure 2) ^17^.

### Quality of evidence

Pooled VE for full and partial dose had moderate quality of evidence. We started at the low quality of evidence because our pooled effects were based on case-control studies (observational studies). No considerable bias was detected using the NOS, all studies were conducted in Africa and directly address review questions, and no heterogeneity or imprecision which was observed to warrant downgrading. However, the evidence was upgraded to moderate quality since the magnitude of the effect was high and consistent throughout the included studies ( Table 2).

**Table 2.** Assessment of quality of evidence for vaccine effectiveness (VE) of partial and full dose.

***Effectiveness of RVA*** ***vaccine in Africa for*** ***partial and full dose***		**Cases** **vaccinated** **(n/Total)**	**Control** **vaccinated** **(n/Total)**	**Adjusted ^1^ VE;** **partial dose** **(OR (95% CI))**	**Adjusted ^1^ VE;** **partial dose** **(OR (95% CI))**	**Overall adjusted ^1^** **VE; partial or full** **dose** **(OR (95% CI))**	**Number of** **participants** **(number of** **studies)**
		335/3468	5948/6519	0.56 (0.43 – 0.72)	0.43 (0.36 – 0.51)	0.47 (0.41 – 0.54)	9987 (13 studies)
***Certainty of*** ***assessment*** ***using GRADE*** ***approach***	**Study** **design**	**Risk of bias**	**Inconsistency**	**Indirectness**	**Imprecision**	**Magnitude of** **effect**	**Overall** **certainty** **of evidence**
	Case control	Low No downgrade ^2^	Low No downgrade ^3^	Low No downgrade ^4^	Low Did not downgrade ^5^	High ^6^Upgraded by 1	⊕⊕⊕Ο MODERATE

Assessment of quality of evidence of vaccine estimates from different age categories. Quality of evidence is graded as high, moderate, low, or very low as a result of downgrading or upgrading the VE estimates. Reasons for downgrading include high risk of bias, inconsistency or heterogeneity, indirectness of the findings, imprecision of the point estimates, and publication bias. The quality of evidence is upgraded if data shows a large effect, a dose-response effect, or if all the plausible residual confounding reduce the demonstrated effect or suggest a spurious effect if no effect was observed. ^1^ Adjusted for age and date of admission in most of the studies. ^2^ No considerable risk of bias was detected using the Newcastle-Ottawa scale (NOS). ^3^ There was no statistical heterogeneity (I ^2^ = 0%), there was also low methodological heterogeneity given that all included studies used similar study design. ^4^ The studies were all conducted in African countries and directly address the review question. ^5^ We did not downgrade for imprecision although some studies had wide confidence intervals. We conducted sensitivity analysis by removing Beres *et al.* 2016 ( *Extended data*: Supplementary File One, Supplementary Figure 3) ^17^, which had widest CI was removed from the meta-analysis and concluded it did not change the pooled estimate. ^6^ The magnitude of effect was high consistently throughout all included studies. Quality of evidence was upgraded by 1.

Our effect estimate based on age categories had low quality of evidence. The evidence was downgraded by one for imprecision due to the few numbers studied used within the different age categories. However, we upgraded the evidence by one since the reported estimates are consistent within the different age categories. No further adjustment was made as no considerable bias was detected by the NOS and all studies were conducted in Africa and directly addressed the review question ( Table 3).

**Table 3.** Assessment of quality of evidence for vaccine effectiveness (VE) by age categories.

***Effectiveness*** ***of RVA*** ***vaccine*** ***in Africa*** ***stratified*** ***by age*** ***categories*** ***dose***	**Cases** **vaccinated** **(n/Total)**	**Control** **vaccinated** **(n/Total)**	**Adjusted ^1^ VE;** **<12 m** **(OR (95% CI))**	**Adjusted ^1^ VE;** **12-23 m** **(OR (95% CI))**	**Adjusted ^1^VE;** **<24 m** **(OR (95% CI))**	**Adjusted ^1^VE;** **12-59 m** **(OR (95% CI))**	**Overall** **adjusted ^1^ VE;** **age categories** **(OR (95% CI))**	**Number of** **participants** **(number of** **studies)**
	3090/4336	5924/6835	0.39 (0.31-0.50)	0.45 (0.29-0.68)	0.44 (0.33-0.57)	0.63 (0.47 – 0.86)	0.46 (0.40 – 0.53)	11171 (21 studies)
***Certainty of*** ***assessment*** ***using GRADE*** ***approach***	**Study design**		**Risk of bias**	**Inconsistency**	**Indirectness**	**Imprecision**	**Magnitude of** **effect**	**Overall** **certainty of** **evidence**
	Case control		Low No downgrade ^2^	Low No downgrade ^3^	Low No downgrade ^4^	Low Downgraded by 1 ^5^	High ^6^Upgraded by 1	⊕⊕ΟΟ Low

Assessment of quality of evidence of vaccine estimates from different age categories. Quality of evidence is graded as high, moderate, low, or very low as a result of downgrading or upgrading the VE estimates. Reasons for downgrading include high risk of bias, inconsistency or heterogeneity, indirectness of the findings, imprecision of the point estimates, and publication bias. The quality of evidence is upgraded if data shows a large effect, a dose-response effect, or if all the plausible residual confounding reduce the demonstrated effect or suggest a spurious effect if no effect was observed. ^1^ Adjusted for age and date of admission in most of the studies. ^2^ No considerable risk of bias was detected using the Newcastle-Ottawa scale (NOS). ^3^ There was statistical heterogeneity ( Figure 4). We conducted sensitivity analysis by dropping Mujuru *et al.* 2019 from analysis because the author stated that VE estimate for this aged group lacked precision and was non-significant. No heterogeneity was observed after dropping this study. There was also low methodological heterogeneity given that all included studies used similar study design. ^4^ The studies were all conducted in African countries and directly address the review question. ^5^ We downgraded for imprecision by 1 due to small number of studies used in some groups, see Figure 3. ^6^ The magnitude of effect was high consistently throughout all included studies. Quality of evidence was upgraded by 1.

## Discussion

Majority of African countries have introduced the Rotarix ^®^ vaccine into their NIP and there is a need to continue making a case for continued vaccination of African children against rotavirus infection. We present pooled VE from case-control studies in the continent showing that full-dose of RV1 vaccine had higher vaccine effectiveness point estimate (VE = 58%, 95% CI: 50-65%) compared to partial dose (VE = 44%, 95% CI: 28-57%). We found that the pooled VE was within range of vaccine efficacy observed during clinical trials in Africa (50–80%).

The confidence intervals (CI) of our VE estimates for full and partial dose were overlapping. Similar evaluation outside Africa by Hungerford *et al.* (2017) shows that full dose of RV1 vaccine had an effectiveness of 89% (95% CI 84-92%) and partial dose had an effectiveness of 62% (95% CI 55-69%) ^38^. Another study evaluating effectiveness in individuals in Latin American and the Caribbean found pooled VE for full dose of RV1 against rotavirus hospitalization was 63.5% (95% CI 39.2-78.0%) when using hospital control and 72.2% (95% CI 60.9-80.2%) ^39^ for community control. Evidently, these estimates of VE are higher than our findings in Africa, hence portraying a similar scenario to the pre-licensure evaluation of rotavirus vaccine efficacy whereby efficacy in high-income countries were higher than low-income countries ^9^. We have also shown evidence of protection against RVA-associated severe diarrhoea beyond the first year of life. All the studies included in this review used enzyme immunoassay (EIA) to identify rotavirus infection. Therefore, the sensitivity and specificity of the tests could not have affected vaccine effectiveness across all studies.

This analysis had some limitations. Primarily, the number of studies reporting effectiveness of RV1 vaccine was still low compared to the number of countries that have introduced RV1. We only used data from the limited number of studies that have been published to date. Including more studies in a future meta-analysis will improve our certainty of the pooled VE estimates from the African continent both for the different age categories and for partial- and full-dose assessment.

In conclusion, we show that RV1 vaccine effectiveness is substantial in Africa and is occurring within the range of efficacy findings observed in clinical trials. The pooled vaccine effectiveness point estimate was lower with a partial dose compared to full dose, thus increased coverage should be encouraged to reap the full benefits of this vaccine. Although the quality of evidence in the age-category based analysis was lower, the data so far appear to support the notion that VE of RV1 is high beyond the first year of life in African children.

## Data availability

### Underlying data

Havard Dataverse: Replication Data for: Effectiveness of Rotarix ^®^ vaccine in Africa in the first decade of progressive introduction, 2009–2019: systematic review and meta-analysis https://doi.org/10.7910/DVN/WJOF7N ^17^.

This project contains the following underlying data:

- Age_categories.tab
- NMurunga_Rotarix_MetaAnalysis_codebook.pdf
- NMurunga_Rotarix_MetaAnalysis_Readme.txt
- Partial_complete_dose.tab
- RV_effectiveness.do

### Extended data

Havard Dataverse: Replication Data for: Effectiveness of Rotarix ^®^ vaccine in Africa in the first decade of progressive introduction, 2009-2019: systematic review and meta-analysis https://doi.org/10.7910/DVN/WJOF7N ^17^.

This project contains the following extended data:

- Supplementary File One.docx
  ◦ Supplementary Text 1. Search strategy of peer-reviewed articles.
  ◦ Supplementary Text 2. Inclusion/exclusion criteria for the systematic review and meta-analysis.
  ◦ Supplementary Figure 1. Estimated pooled vaccine effectiveness for complete dose of RV1 against laboratory-confirmed rotavirus infection with Armah *et al.* 2016 excluded.
  ◦ Supplementary Figure 2. Estimated pooled vaccine effectiveness for complete dose of RV1 against laboratory-confirmed rotavirus infection stratified by age categories after excluding Mujuru *et al.* 2019.
  ◦ Supplementary Figure 3. Estimated pooled vaccine effectiveness for complete dose of RV1 against laboratory-confirmed rotavirus infection with Beres et al 2016 excluded.
- Supplementary File Two.tab (data collection form)
- Supplementary File Three.pdf (Risk of Bias Assessment for All Case Control Studies Included in Vaccine Effectiveness Evaluation using Newcastle Ottawa Scale).

### Reporting guidelines

Harvard Dataverse: PRISMA checklist for ‘Effectiveness of Rotarix® vaccine in Africa in the first decade of progressive introduction, 2009–2019: systematic review and meta-analysis’ (Supplementary File One, Table 1), https://doi.org/10.7910/DVN/WJOF7N ^17^.

Data are available under the terms of the Creative Commons Attribution 4.0 International license (CC-BY 4.0).
