# Supplementary material for: Effectiveness of Rotarix ® vaccine in Africa in the first decade of progressive introduction, 2009-2019:  systematic review and meta-analysis
Source: Wellcome Open Res. 2020 Sep 24;5:187. Originally published 2020 Aug 6. [Version 2] doi: 10.12688/wellcomeopenres.16174.2 (PMC7658728; doi:10.12688/wellcomeopenres.16174.2)
Supplement: Peer review history

# Peer review: Reviewer response for version 2

**DOI:** 10.21956/wellcomeopenres.17919.r41126

**Published:** 2020-11-11

**Authors:** Julia M Baker

**Affiliations:** 1 Department of Epidemiology, Rollins School of Public Health, Emory University, Atlanta, Georgia, USA

In this study, Murunga *et al.* examine two important questions: 1) the full vs. partial dose vaccine effectiveness of RV1 in African countries and 2) effectiveness across age groups. Investigation into these questions will further guide vaccination strategies in a setting where the burden of rotavirus is greatest. Overall, this is a thoroughly conducted study with several relevant sensitivity analyses and quality assessments. The manuscript well describes the results and interpretation of findings.

 Major comments:

- Please provide more detail regarding your random effects model. Did you use a random intercept, random slopes, or both? How did you account for variations in effect size?

Minor comments:

- In Table 1, the "Partial dose Full dose" heading should be separated into two columns to correspond with the rows below.
- In Table 1, some of the studies indicate age groups greater than a certain age (e.g. >=6m). Please indicate the maximum age for these as well. Were they all <60m?
- Please clarify what the grey shaded areas represent in Figure 2.
- It seems worth elaborating more on the age stratified results in the discussion section (while noting that the quality of the evidence is poor). These results may be helpful for addressing questions about potential waning immunity.

Are the rationale for, and objectives of, the Systematic Review clearly stated?

Yes

Is the statistical analysis and its interpretation appropriate?

Yes

Are sufficient details of the methods and analysis provided to allow replication by others?

Partly

Are the conclusions drawn adequately supported by the results presented in the review?

Yes

Reviewer Expertise:

Vaccine effectiveness evaluation.

I confirm that I have read this submission and believe that I have an appropriate level of expertise to confirm that it is of an acceptable scientific standard.

# Peer review: Reviewer response for version 2

**DOI:** 10.21956/wellcomeopenres.17919.r40602

**Published:** 2020-10-16

**Authors:** Miguel O'Ryan, Sergio George

**Affiliations:** 1 Faculty of Medicine, University of Chile, Santiago, Chile; 2 Programa de Microbiología, ICBM, Facultad de Medicina, Universidad de Chile, Santiago, Chile; 3 Instituto Milenio de Inmunidad e Inmunoterapia, Facultad de Medicina, Universidad de Chile, Santiago, Chile

All comments have been addressed

Are the rationale for, and objectives of, the Systematic Review clearly stated?

Yes

Is the statistical analysis and its interpretation appropriate?

I cannot comment. A qualified statistician is required.

Are sufficient details of the methods and analysis provided to allow replication by others?

Yes

Are the conclusions drawn adequately supported by the results presented in the review?

Yes

Reviewer Expertise:

Pediatric infectious diseases, vaccines, enteric infections

We confirm that we have read this submission and believe that we have an appropriate level of expertise to confirm that it is of an acceptable scientific standard.

# Peer review: Reviewer response for version 1

**DOI:** 10.21956/wellcomeopenres.17758.r39864

**Published:** 2020-08-18

**Authors:** Miguel O'Ryan, Sergio George

**Affiliations:** 1 Faculty of Medicine, University of Chile, Santiago, Chile; 2 Programa de Microbiología, ICBM, Facultad de Medicina, Universidad de Chile, Santiago, Chile; 3 Instituto Milenio de Inmunidad e Inmunoterapia, Facultad de Medicina, Universidad de Chile, Santiago, Chile

Nickson Murunga and collaborators present a comprehensive review and meta-analysis, pooling data from identified studies to determine effectiveness of RV1 (Rotarix®) in Africa during the last 10 years.

 The manuscript is well-written and precise (with some grammar mistakes that can be reviewed) with an adequate description of the search strategy and analysis process, although with the characteristic technical descriptions that requires expertise for appropriate evaluation. The search leads to a rather small (unfortunately not more data is available) but reasonably robust number of overall participants. The study should be reviewed by an expert in meta-analysis methods to assure that the methods used in this study are correct.

**Specific comments:**

**Introduction**

- Authors should explain and detail differences between terms “effectiveness” and “impact”. As mentioned in background, RVA vaccine implementation in Africa was based on expected impact on disease, despite clinical trials showed lower efficacy compared to industrialized countries. Studies published post vaccine introduction are focused on its impact and/or effectiveness, and this review focuses only in those studies assessing effectiveness. It is possible to assume that “impact” refers to overall effect of vaccine in a population (which includes vaccinated and non-vaccinated subjects) and “effectiveness” refers to direct effect of vaccine (only in vaccinated subjects). However, in order to be understandable to general scientific community authors should specify this difference. 

**Methods:**

- The fact that the term “impact” was included in the search strategy followed by exclusion of 28 studies focusing on impact of vaccine on diarrhea hospitalization and diarrhea associated mortality is unclear.
- Authors should specify if “partial dose” refers to 1 dose of Rotarix, and “complete dose” to 2 doses in all studies included in this review.
- There is no mention of diagnostic tests used to identify rotavirus infection in the different studies. As sensitivity and specificity differs between tests, this variable may affect effectiveness results in different studies.

**Results: **

- Table 1 and 2 are excessively redundant (they can be merged).
- Table 3 is not required; it can be reduced to one sentence highlighting the only different study (Abeid *et al.*).
- The reasoning provided for the sensitivity analysis are unclear; is this standard methodology?
- Tables 4 and 5 are somewhat difficult to understand, should be better explained.

**Discussion:**

- More caution in the full dose vs partial dose conclusion as there is no significant difference.
- Authors compare results of this meta-analysis with a study performed in Latin America which used both hospital and community controls. This lead to ask if all studies included in this meta-analysis used only hospitalized controls, and if so, was it because studies from a non-hospital setting were not found in the literature search?
- “…falling within range” may be misleading, better “within range”.
- Evidently, this estimates, should be these estimates.

Are the rationale for, and objectives of, the Systematic Review clearly stated?

Yes

Is the statistical analysis and its interpretation appropriate?

I cannot comment. A qualified statistician is required.

Are sufficient details of the methods and analysis provided to allow replication by others?

Yes

Are the conclusions drawn adequately supported by the results presented in the review?

Yes

Reviewer Expertise:

Pediatric infectious diseases, vaccines, enteric infections.

We confirm that we have read this submission and believe that we have an appropriate level of expertise to confirm that it is of an acceptable scientific standard, however we have significant reservations, as outlined above.

# Response

**Published:** 2020-09-07

**Authors:** Nickson Murunga

**Affiliations:** Kenya Medical Research Institute (KEMRI)-Wellcome Trust Research Programme, Kenya

**Introduction Section**

1. Authors should explain and detail differences between terms “effectiveness” and “impact”.As mentioned in background, RVA vaccine implementation in Africa was based on expected impact on disease, despite clinical trials showed lower efficacy compared to industrialized countries. Studies published post vaccine introduction are focused on its impact and/or effectiveness, and this review focuses only in those studies assessing effectiveness. It is possible to assume that “impact” refers to overall effect of vaccine in a population (which includes vaccinated and non-vaccinated subjects) and “effectiveness” refers to direct effect of vaccine (only in vaccinated subjects). However, in order to be understandable to general scientific community authors should specify this difference.

**Authors Response: **Thanks for pointing this out, we have clarified the difference in the revised manuscript.

2.  The fact that the term “impact” was included in the search strategy followed by exclusion of 28 studies focusing on impact of vaccine on diarrhea hospitalization and diarrhea associated mortality is unclear.

**Authors Response: **Impact was included in our search strategy because during our screening we realized some papers titled reporting on the impact of rotavirus vaccine had within their results section an estimate of local vaccine effectiveness. Excluding impact in our search strategy will have excluded some of the papers that have reported on vaccine effectiveness. An example is a paper by Plats-Mills et al. 2017 “Impact of Rotavirus Vaccine Introduction and Post introduction Etiology of Diarrhea Requiring Hospital Admission in Haydom, Tanzania, a Rural African Setting”.

3. Authors should specify if “partial dose” refers to 1 dose of Rotarix, and “complete dose” to 2 doses in all studies included in this review.

**Authors Response: **Thank you for pointing this out. We have added this clarification in Tables and Figure 2 legends.

4. There is no mention of diagnostic tests used to identify rotavirus infection in the different studies. As sensitivity and specificity differs between tests, this variable may affect effectiveness results in different studies.

**Authors Response: **Thank you for pointing this out. We have included this in our revised version.

**Results Section**

****

5.     Table 1 and 2 are excessively redundant (they can be merged).

**Authors Response: **We have merged the two tables. Included in our revised manuscript.

6.     Table 3 is not required; it can be reduced to one sentence highlighting the only different study (Abeid et al.).

**Authors Response:** Thank you for the observation. This has been amended in our revised manuscript.

7. The reasoning provided for the sensitivity analysis are unclear; is this standard methodology?

**Authors Response: **Yes. This is a standard methodology. Sensitivity analysis is one of the strategies for addressing heterogeneity see https://training.cochrane.org/handbook/current/chapter-10#section-10-11.

8.     Tables 4 and 5 are somewhat difficult to understand, should be better explained.

**Authors Response: ** Thanks for this observation. More explanation is provided in our revised manuscript.

**Discussion:**

****

9.     More caution in the full dose vs partial dose conclusion as there is no significant difference.

**Authors Response: **This has been rephrased in our revised manuscript.

10.  Authors compare results of this meta-analysis with a study performed in Latin America which used both hospital and community controls. This lead to ask if all studies included in this meta-analysis used only hospitalized controls, and if so, was it because studies from a non-hospital setting were not found in the literature search?

**Authors Response: **Thank you for noting this. From our literature search, only one study from Malawi ( https://www.ncbi.nlm.nih.gov/pmc/articles/PMC4374102/) used both hospital and community control. A similar magnitude of vaccine effectiveness was established for both controls. Therefore, only hospital control was included in this meta-analysis.

11.  “…falling within range” may be misleading, better “within range”.

**Authors Response**: Corrected.

12.  Evidently, this estimate, should be these estimates.

**Authors Response: **Corrected **.**
